# Application of thermosensitive-hydrogel combined with dental pulp stem cells on the injured fallopian tube mucosa in an animal model

**DOI:** 10.3389/fbioe.2022.1062646

**Published:** 2023-01-06

**Authors:** Lihua Luo, Qunyan Zhu, Yejian Li, Fengting Hu, Jiangtao Yu, Xiangyan Liao, Zhenjie Xing, Yan He, Qingsong Ye

**Affiliations:** ^1^ School and Hospital of Stomatology, Wenzhou Medical University, Wenzhou, Zhejiang, China; ^2^ The First Affiliated Hospital of Wenzhou Medical University, Wenzhou, Zhejiang, China; ^3^ Tianyou Hospital, Wuhan University of Science and Technology, Wuhan, China; ^4^ Center of Regenerative Medicine, Renmin Hospital of Wuhan University, Wuhan, China

**Keywords:** dental pulp stem cells, fallopian tube injury, hydrogel, immunoregulation, repair

## Abstract

**Objectives:** Fallopian tube (FT) injury is an important factor that can lead to tubal infertility. Stem-cell-based therapy shows great potential for the treatment of injured fallopian tube. However, little research has shown that mesenchymal stem cells (MSCs) can be used to treat fallopian tube damage by *in situ* injection. In this study, we *in situ* transplanted PF127 hydrogel encapsulating dental pulp stem cells (DPSCs) into the injured sites to promote the repair and regeneration of fallopian tube injury.

**Materials and methods:** The properties of dental pulp stem cells were evaluated by flow cytometry, immunofluorescence analysis, and multi-differentiation detection. The immunomodulatory and angiogenic characteristics of dental pulp stem cells were analyzed on the basis of the detection of inflammatory factor expression and the formation of capillary-like structures, respectively. The biocompatibility of PF127 hydrogel was evaluated by using Live/Dead and CCK-8 assays. The effects of PF127 hydrogel containing dental pulp stem cells on the repair and regeneration of fallopian tube injury were evaluated by histological analysis [e.g., hematoxylin and eosin (H&E) and Masson’s trichrome staining, TUNEL staining, immunofluorescence staining, and immunohistochemistry], Enzyme-linked immunosorbent assay (ELISA), and RT-PCR detections.

**Results:** Dental pulp stem cells had MSC-like characteristics and great immunomodulatory and angiogenic properties. PF127 hydrogel had a thermosensitive feature and great cytocompatibility with dental pulp stem cells. In addition, our results indicated that PF127 hydrogel containing dental pulp stem cells could promote the repair and regeneration of fallopian tube damage by inhibiting cell apoptosis, stimulating the secretion of angiogenic factors, promoting cell proliferation, modulating the secretion of inflammatory factors, and restoring the secretion of epithelial cells.

**Conclusion:** In this study, our results reported that *in situ* injection of PF127 hydrogel encapsulating dental pulp stem cells into the injured sites could provide an attractive strategy for the future treatment of fallopian tube injury in clinical settings.

## 1 Introduction

The World Health Organization has reported that the infertility rate is more than 15% among women of reproductive age worldwide, and infertility should be regarded as a public health problem (Adegbola and Akindele 2013). Tubal infertility is a common cause of female infertility, accounting for 30%–35% (Zou et al., 2014). Many influencing factors, including infection, fallopian tube (FT) surgery, ectopic pregnancy, and endometriosis, may cause salpingitis or pelvic inflammation with FT mucosa damage and structural collapse, which are important indicators of tubal infertility (Li et al., 2017). At present, tubal infertility shows a yearly rising trend because of the popularity of sexually transmitted diseases and the repetitive infection of FT (Luo et al., 2015; Almasry et al., 2018).

Recently, the principal curing methods for tubal infertility include laparoscopic surgery and antibiotic therapy in clinical settings. However, the repetitive attachment of FT after surgery and the occurrence of antibiotic-resistant strains of the long-term use of antibiotics can lead to failure treatment of tubal infertility (Liao et al., 2019). Therefore, new therapeutic strategies have been required to repair the injury of FT and restore the reproduction function of FT, thereby reducing the incidence of tubal infertility.

Mesenchymal stem cells (MSCs) have pluripotent characteristics, and they can be induced to differentiate into many types of cells, which could provide tissue-reconstructing cells in the damaged sites. In addition, MSCs have shown strong paracrine effects by secreting several growth factors and providing immunosuppressive and anti-inflammatory actions in stem-cell-based therapies (Liao et al., 2019). Thus, MSC-based therapy can provide a novel strategy for the repair and regeneration of injured FT.

Dental pulp stem cells (DPSCs), as a kind of MSCs, can be readily cultured from the dental pulps which are isolated from impacted third molars, exfoliated deciduous teeth, permanent and primary teeth, and supernumerary teeth, without extra harm and invasive surgical procedures (Luo et al., 2018; Luo et al., 2021). DPSCs possess all MSCs-like properties, e.g. multi-differentiation potential and immunomodulatory properties, and high proliferative and self-renewal capacities (Luo et al., 2018). In addition, the capacity of DPSCs to secrete growth factors is higher than other tissue-derived MSCs such as bone-marrow-derived mesenchymal stem cells (BM-MSCs). Meanwhile, DPSCs have a higher proliferation rate and greater immunomodulatory properties than BM-MSCs (Lan et al., 2019). Therefore, DPSCs have become an attractive seeded cell in stem-cell-based therapies. Moreover, we have confirmed an angiogenic characteristics of DPSCs’ in our previous work (Luo et al., 2018), where DPSCs participated in the formation of new blood vessels and enhanced the blood supply around injured sites. Angiogenesis is an important influencing factor for the treatment of FT injury.

Although DPSCs have outstanding MSC-like properties, using DPSCs alone can hardly receive ideal restoration of reproduction function after FT injury because of their poor cellular density and survival rate in damaged sites. As shown in previous studies, hydrogels have a 3D network structure and great porosity, which can be used to accommodate cells and provide a suitable microenvironment for cell survival and growth (Zhu and Marchant 2011). Our previous studies indicated that the thermosensitive Pluronic F-127 (PF127, also known as poloxamer 407) hydrogel, which has a solution state at 4°C and a hydrogel state at human body temperature, had a porous structure and great cytocompatibility, and it could be a great cell carrier in tissue engineering and regenerative medicine (Albashari et al., 2020). Furthermore, the controllable degradability of PF127 hydrogel could provide an appropriate microenvironment for cellular adhesion, growth and proliferation (Supplementary Figure S5). Thus, in this work, we used PF127 as the scaffold and transplanted PF127 hydrogel containing DPSCs into the damaged site to evaluate its effects on the repair and regeneration of FT injury.

## 2 Materials and methods

### 2.1 Ethics and animals

Twenty-four female New Zealand white rabbits (weighing 2,000 ± 250 g) were provided by the Animal Experimental Center of Wenzhou Medical University and housed with sufficient water and food in animal cages for 14 days before surgery. All experimental procedures were performed under the regulations of the committee of the Institutional Animal Care and the Ethics Committee of Wenzhou Medical University (wydw 2019-0949).

### 2.2 Isolation, culture, and identification of DPSCs

The isolation and culture procedures of DPSCs were performed as previously described (Luo et al., 2021). In brief, dental pulp tissues, which were collected from the tooth, were cut into small pieces and digested using the mixture of collagenase type I (Gibco, United States) and dispase (Sigma, Germany) for 30 min at 37°C in a 5% CO_2_ cell incubator. Then, the pulp tissue was suspended and cultured using α-modified Eagle’s medium (α-MEM, Gibco, United States) supplemented with 20% fetal bovine serum (FBS, Gibco, United States), 100 μg/ml of streptomycin, and 100 U/mL of penicillin (Gibco, United States) at 37°C in a 5% CO_2_ cell incubator. The culture medium was replaced every 3 days. MSC-like characteristics of DPSCs were identified by flow cytometry, immunofluorescence analysis, and multi-differentiation detection. Flow cytometry was executed using human primary antibodies, including CD19, CD166, CD73, CD90 (BD Pharmingen™, United States), CD34, and HLA-DR (BioLegend, United States), and the data were evaluated using a CytoFLEX flow cytometer (Beckman Coulter, California, United States). The immunophenotype of DPSCs was analyzed using primary antibodies against CD44 (1:400, Abcam, Cambridge, United Kingdom), and the immunofluorescence of DPSCs was observed using a fluorescence microscope (Eclipse 80i, Nikon, Japan). Based on the procedures described in our previous work (Albashari et al., 2020; Luo et al., 2021), the multipotent property of DPSCs was analyzed by osteogenic, adipogenic, and chondrogenic differentiation with alizarin red S staining, Oil Red O staining, and Alcian blue staining, respectively. Images were observed using a light microscope (TS100, Nikon).

### 2.3 Effects of DPSCs on murine macrophage RAW264.7 cells

The effects of DPSCs on murine macrophage RAW264.7 cells were evaluated by performing a macrophage-DPSCs coculture experiment as previously described (Németh et al., 2009; Chen et al., 2019). First, the mouse monocyte-macrophage cells (RAW 264.7, ATCC, United States) were cultured in 24-well plates at a concentration of 2 × 10^5^ cells/well. After incubation for 24 h, the supernatants were removed, and the cells were treated with lipopolysaccharide (LPS, Sigma; 1 μg/ml) for 24 h. Then, DPSCs (2 × 10^4^ cells/well), which were cultured in insert membranes of the transwell system (.4 μm pore size, Corning), were transferred to the wells. After incubation for another 24 h, the supernatants were collected and stored at −20°C for further use. The expression of tumor necrosis factor-alpha (TNF- α ) and interleukin-10 (IL-10) of challenged cells and supernatants was assessed by immunofluorescence staining and Enzyme-linked immunosorbent assay (ELISA) (R&D Systems), respectively.

### 2.4 Evaluation of angiogenic characteristics of DPSCs

To evaluate the angiogenic characteristics of DPSCs, following *in vitro* experiment was performed to assess the formation of capillary-like structures. 96-well plates were pre-coated with gelatin methacryloyl (GelMA) hydrogel as previously described (Chen et al., 2012). In brief, DPSCs and human umbilical vein endothelial cells (HUVECs, ATCC, United States) were plated onto hydrogel-treated 96-well plates and cultured with an endothelial cell growth medium-2™ (EGM-2™, Lonza Bioscience, Switzerland) supplemented with 2% FBS, .4% hFGF-B, .1% VEGF, .1% hEGF, .1% R3-IGF-1, .1% Heparin, .1% ascorbic acid, .1% gentamicin/amphotericin-B, and .04% hydrocortisone. The cultured medium was changed every 2 days. After differentiation for 7 days, the cells were fixed with 4% PFA and treated with Phalloidin-TRITC (1:200, Solarbio, China), and the nuclei were stained by 4′6-Diamidino-2-phenylindole-dihydrochloride (DAPI, Beyotime Institute of Biotechnology, Shanghai, China). The images were taken using a fluorescence microscope (Eclipse 80i, Nikon, Japan), and the vascular tube length and number were calculated using NIS-Elements AR 3.1 (Nikon).

### 2.5 Preparation of PF127 hydrogel

PF127 powder was slowly added to the complete α-MEM (containing 20% FBS, 100 μg/ml of penicillin, and 100 μg/ml of streptomycin) to prepare a 20% (w/v) solution. The solution was placed on a shaking table at 4°C for 24 h to ensure that the powder was fully dissolved. Then, the PF127 solution was filtered by using a .22 μm pore size bottle-top filter and stored at 4°C for future use. Our previous study confirmed that the PF127 solution could preserve a solution state at 4°C and transform a hydrogel state at body temperature (Albashari et al., 2020).

### 2.6 Cytocompatibility of PF127 hydrogel

The biocompatibility of PF127 hydrogel was evaluated using a Live/Dead Viability/Cytotoxicity Kit (Invitrogen, CA, United States) and Cell Counting Kit-8 (CCK-8, Dojindo Molecular Technologies) assay. In brief, the cultured DPSCs were trypsinized and resuspended in PF127 solution on ice. Then, 200 μl of cell-PF127 mixture solution containing 5 × 10^4^ cells/ml was added to a 48-well plate. After seeding, the plate was kept at 37°C in 5% CO_2_ for 5 min to induce gel formation. The fresh medium was then added to each well, and the plate was transferred to a cell incubator. After being incubated for 1, 3, and 5 days, the Calcein-AM/pyridine iodide reagent mixture solution was added to the plates and incubated in the dark at 37°C for 10 min and observed using a fluorescence microscope (Eclipse 80i, Nikon, Japan). 10% CCK-8 solution was added to each well and transferred to a cell incubator for another 2 h to quantitatively analyze the cell proliferation activity. Absorbance at 450 nm was measured using an absorbance microplate reader (Varioskan LUX, Thermo Fisher Scientific).

### 2.7 Animal model establishment

All experiments were performed under sterile conditions. Female rabbits were anesthetized by a mixture of 2% isoflurane in 70% nitrous oxide and 30% oxygen and maintained with 1% isoflurane *via* a face mask. Then the skin was prepared, and a 4–5 cm incision was made to expose the FT and ovaries. The junction between the FT and the uterus was clamped with an arterial clip. .1 ml of absolute ethanol was injected into the FT using a 1 ml syringe. After 2 min, the arterial clip was loosened, and the FT was wiped around using a moist gauze and sutured. After 4 h, the HE results indicated that the FT had evident damage, and the structure of mucosa folds collapsed (Supplementary Figure S1), indicating that the animal model of FT injury was successfully constructed.

### 2.8 Experimental animals and grouping

Twenty four female white rabbits were randomly assigned to four groups: 1) sham group without any treatment; 2) FT injury (FTI) group that directly injected saline into the FT after the effect of anhydrous alcohol for 4 h; 3) DPSCs group that received an intravenous injection of DPSCs at the ear margin after the effect of anhydrous alcohol for 4 h; 4) PF127-encapsulated DPSCs (DPSCs-PF127) group that injected the mixture of DPSCs and PF127 into the FT after the effect of anhydrous alcohol for 4 h. After treatment for 4 weeks, the rabbits were euthanized by intraperitoneal injection of 3% pentobarbital sodium and the samples were collected and used for future analysis.

### 2.9 Histopathologic evaluation

The FT specimens were fixed in 4% formaldehyde, embedded in paraffin, sectioned into slides, stained with hematoxylin and eosin (H&E) and Masson’s trichrome, and recorded using an optical microscope (ECLIPSE 80i, Nikon, Tokyo, Japan).

### 2.10 TdT-mediated dUTP nick-end labeling (TUNEL) staining

The TUNEL apoptosis assay kit (Beyotime, Nantong, Jiangsu, China) assay was performed as previously described (Zhu et al., 2020). In brief, the sections were dewaxed in xylene and rehydrated with gradient ethanol. Then, the sections were treated with TUNEL-FITC (1:200), and the nuclei were stained with DAPI for 10 min. Images were taken using a fluorescence microscope (Eclipse 80i, Nikon, Japan).

### 2.11 Immunofluorescence and immunohistochemistry

Immunofluorescence was performed to assess the primary antibody of VEGF-A and KI67 according to the manufacturer’s procedures. The sections were treated with primary antibodies, namely, VEGF-A (1:200, Abcam, Cambridge, United Kingdom) and KI67 (1:200, Abcam, Cambridge, United Kingdom), overnight. Then, the sections were incubated with fluorogenic secondary antibodies for 1 h at 37°C. Finally, the sections were washed with PBS and incubated with DAPI for 10 min. For immunohistochemistry, the sections were treated with a primary antibody, namely, Vimentin (VIM; 1:200, Abcam, Cambridge, United Kingdom), overnight at 4°C and then incubated with horseradish-peroxidase-conjugated secondary antibody for another 2 h at 37°C. Then, the sections were developed using 3,3′-diaminobenzidine. All sections were observed using a fluorescence microscope (Eclipse 80i, Nikon, Japan).

### 2.12 ELISA

The concentrations of TNF-α and IL-10 in serum were measured using TNF-α (CSB-E06998Rb, CUSABIO Biotech Co., Ltd., Wuhan, China) and IL-10 (CSB-E06897Rb, CUSABIO Biotech Co., Ltd., Wuhan, China) ELISA kits. The blood of rabbits was collected; the serum was obtained after centrifugation, and the serum level of inflammatory cytokines was detected in accordance with the manufacturer’s protocols. All of the samples were repeated.

### 2.13 Gene expression analysis

After 4 weeks, the FT was collected. Total RNA was extracted from 50 mg of FT using a TRIzol reagent (Invitrogen, CA, United States). One microgram of RNA was reverse transcribed into cDNA using a PrimeScript™ RT reagent kit gDNA Eraser (RR047A, TaKaRa BIO Inc., Kusatsu, Shiga, Japan). Real-time PCR was performed using the SYBR Premix EX TaqTM II. cDNA was amplified using the primer sequences shown in Table 1. The PCR cycling parameters used were as follows: 95°C for 10 s, annealed at 95°C for 5 s and extended at 60°C for 30 s for 40 cycles, followed by thermal denaturation. The mRNA expression was evaluated on the basis of SYBR green fluorescence for 18 s as the endogenous control.

**TABLE 1:** Forward and reverse primer sequences.

Genes	Primer sequence (5′–3′)	
	Forward	Reverse
18S	GAA​TTC​CCA​GTA​AGT​GCG​GGT​CAT​A	CGA​GGG​CCT​CAC​TAA​ACC​ATC
OVGP	GGA​TGT​CTG​AAG​CAC​CCA​GAG​GT	AGG​TCA​TCG​TCA​TCT​TGC​CAG​GG
IL10	GCC​AAG​CCT​TGT​CGG​AGA​TGA​TC	CTG​CTC​CAC​TGC​CTT​GCT​CTT​G
KI67	ATG​TTC​CAT​TTC​ATC​AGC​CA	TTT​AAA​TCG​CTC​CTC​CAT​CC
Caspase-3	TGT​AAA​TGC​AGC​AAA​CCT​CG	GAC​TCC​TTC​ATC​ACC​GTG​GC

### 2.14 Statistical analysis

Graph pad 7.0 statistics was used to process the data, and the results were presented as means ± standard errors. Statistical differences were obtained by Student’s *t-*test and one-way analysis of variance, followed by Tukey’s test or Dunnett *post hoc* test. *p* < .05 was considered as significant differences.

## 3 Results

### 3.1 DPSCs expressed MSC-like markers and possessed multipotency

The dental pulp is a soft connective tissue containing mesenchymal tissues, which is located in the medullary cavity of the tooth. DPSCs were extracted from the dental pulp, and the procedure for obtaining dental pulp tissues is shown in Supplementary Figure S2A. DPSCs had great cell proliferation capacity, and they can complete the whole primary culture for about 20 days (Supplementary Figure S2B). DPSCs possessed MSC-like properties and positively expressed MSC-like surface markers, including CD166, CD73, CD90, and CD44 (Supplementary Figures S3A, B), but they negatively expressed hematopoietic lineage markers, including CD19, CD34, and HLA-DR (Supplementary Figure S3A). Moreover, DPSCs had an excellent capacity for multilineage differentiation, and they could form mineralized nodules by positive staining of alizarin red S in the induced differentiation of osteoblasts. DPSCs were also induced to differentiate into adipogenesis with the formation of lipid droplets, which were stained with oil red O. In addition, DPSCs could differentiate into chondrocytes and show the evident staining of Alcian Blue (Supplementary Figure S3C).

### 3.2 DPSCs inhibited the expression of a pro-inflammatory factor (TNF-α) and enhanced the expression of an anti-inflammatory cytokine (IL-10)

**FIGURE 1:**
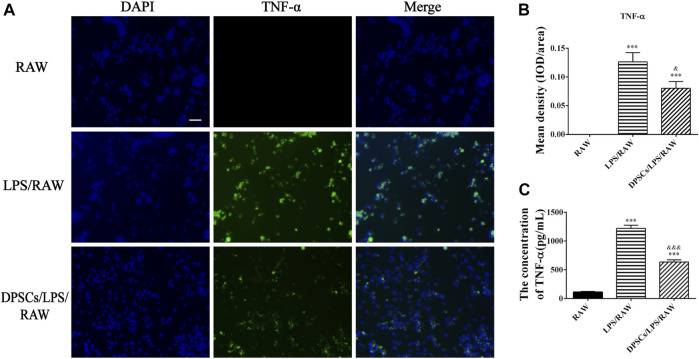
The expression of TNF-α in DPSCs co-cultured with RAW264.7 cells *in vitro*. **(A)** Immunofluorescence staining of TNF-α. Scale bar: 50 μm. **(B)** Quantification of the fluorescence intensity of TNF-α. ***p < .001 versus the RAW group. ^&^ *p* < .05 versus the LPS/RAW group. **(C)** Comparison of the supernatants TNF-α concentration among groups. ***p < .001 versus the RAW group. ^&&&^ *p* < .001 versus the LPS/RAW group.

**FIGURE 2:**
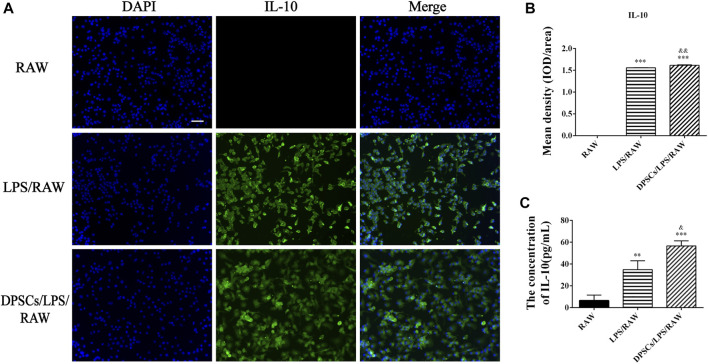
The expression of IL-10 in DPSCs co-cultured with RAW264.7 cells *in vitro*. **(A)** Immunofluorescence staining of IL-10. Scale bar: 50 μm. **(B)** Quantification of the fluorescence intensity of IL-10. ***p < .001 versus the RAW group. ^&&^ *p* < .01 versus the LPS/RAW group. **(C)** Comparison of the supernatants IL-10 concentration among the groups. **p < .01, ***p < .001 versus the RAW group. ^&^ *p* < .05 versus the LPS/RAW group.

The effects of DPSCs on the secretion of inflammatory factors in LPS-induced RAW264.7 cells were evaluated by immunofluorescence staining and ELISA. The results indicated that the expression of TNF-α and IL-10 was upregulated in macrophages by LPS stimulation for 24 h. The expression level of TNF-α was reduced in the presence of DPSCs (Figures 1A, B), whereas the expression of IL-10 was significantly increased (Figures 2A, B). As shown in Figures 1C, 2C, the results indicated that the level of TNF-α and IL-10 in supernatants was also increased after LPS induction for 24 h. In addition, DPSCs could downregulate the expression of TNF-α and upregulate the expression of IL-10, and they showed a significant difference compared with the LPS-induced one.

### 3.3 DPSCs promoted the formation of capillary-like structures

**FIGURE 3:**
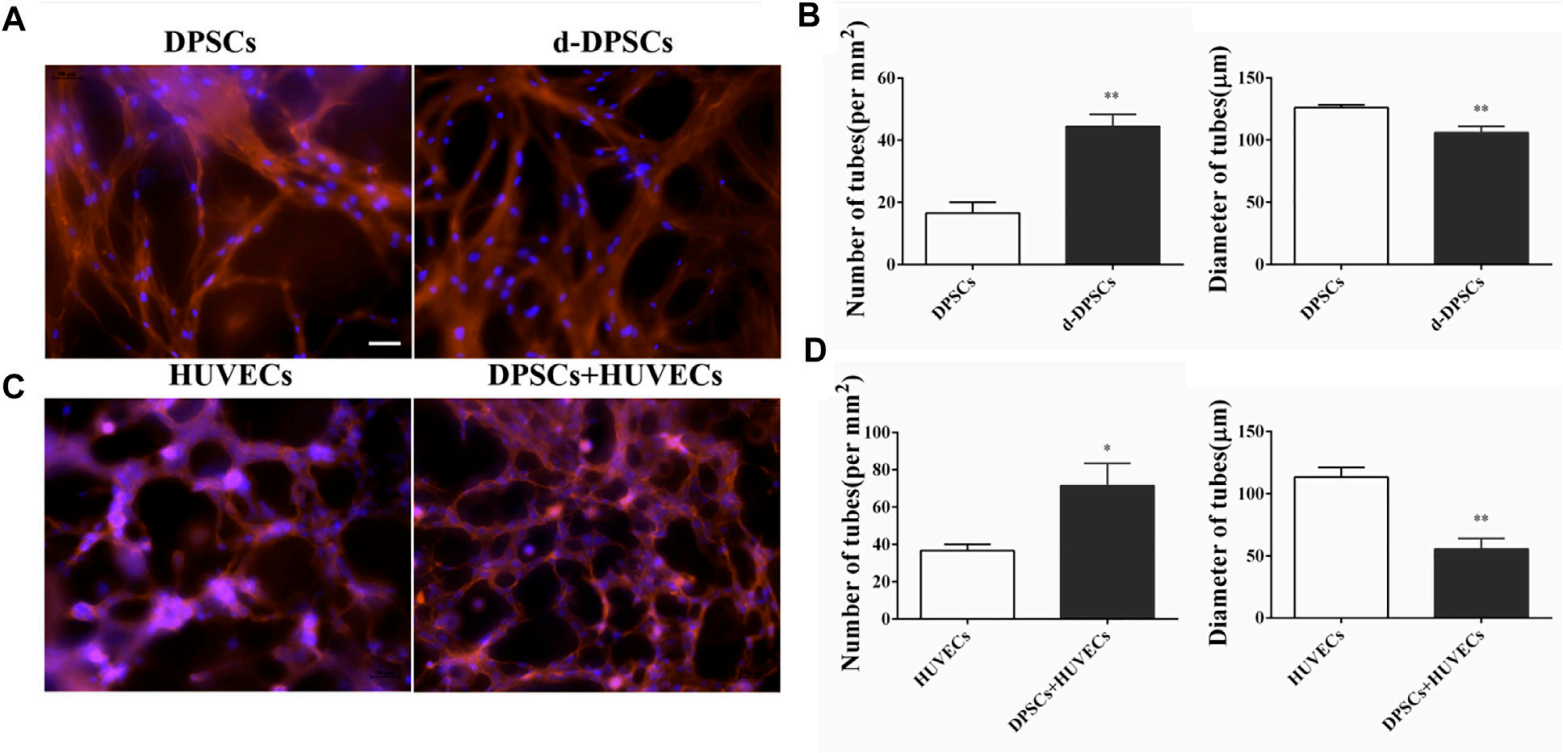
The effects of DPSCs on the formation of vessel-like structures. **(A)** The angiogenic potential of DPSCs by directly induced by EGM-2 TM complete culture medium. **(B)** The number and diameter of the formed tubes between the DPSCs group and the differentiated DPSCs (d-DPSCs). **p < .01 versus the DPSCs group. **(C)** The effects of DPSCs on the tube formation of HUVECs by the cocultured system. **(D)** The number and diameter of the formed tubes between the HUVECs group and the HUVECs cocultured with DPSCs group (DPSCs+HUVECs).*p < .05, **p < .01 versus the HUVECs group. Scale bar: 50 μm.

The angiogenic characteristics of DPSCs were evaluated by tubular network formation. The directly induced DPSCs (d-DPSCs), which were 3D cultured with GelMA hydrogel in the completed EGM-2™ medium, could form capillary-like networks. In addition, the number and diameter of closed networks were higher in d-DPSCs than in DPSCs cultured with α-MEM medium, and the difference was statistically significant (Figures 3A, B). Moreover, our results indicated that DPSCs could enhance the capillary-like structure formation of HUVECs using the indirectly cocultured system. Meanwhile, the number and length of capillary-like structures were higher in HUVECs cocultured with DPSCs than in HUVECs cultured alone (Figures 3C, D).

### 3.4 PF127 hydrogel had a thermosensitive property and great biocompatibility

**FIGURE 4:**
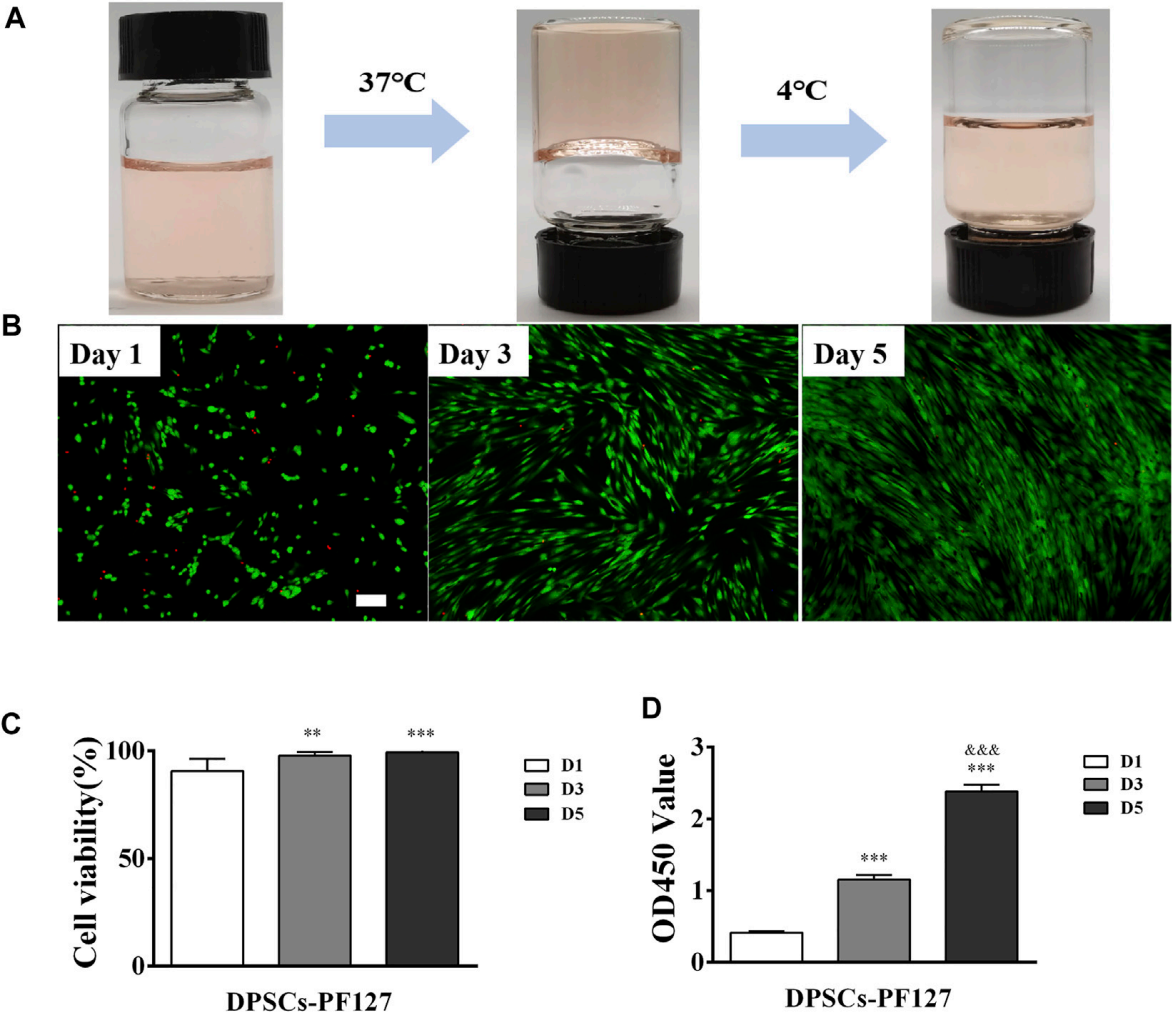
The biocompatibility of PF127 hydrogel. **(A)** The thermosensitive property of PF127. **(B)** The images of Dead/Live assay of DPSCs encapsulated into the PF127 hydrogel after 1, 3 and 5 days, Scale bar: 200 μm. **(C)** Statistical analysis of the cell viability. The ratio of green to green plus red cells provided the percentage of cell viability. **p < .01, ***p < .001 versus the Day 1. **(D)** The proliferation activity of DPSCs encapsulated into the PF127 hydrogel after 1, 3 and 5 days were measured using Cell Counting Kit-8. ***p < .001 versus the Day 1, ^&&&^ *p* < .001 versus the Day 3.

As shown in Figure 4A, PF127 hydrogel demonstrated a thermosensitive characteristic. It was clear and liquid solution when it was cold and transited to a homogeneous semi-solid hydrogel status at 37°C. Its gelation time was 80 ± 5 s. The biocompatibility of PF127 hydrogel containing DPSCs was evaluated using Live/Dead and CCK-8 assays. As for the Live/Dead assay, the results indicated that PF127 hydrogel had great cytocompatibility. After being cocultured with DPSCs, most of the cells were alive and stained with green from day 1 to day 5. In addition, the ratio of live cells had no difference between day 3 and day 5, but all of them were higher than that of day 1 (Figures 4B, C). The proliferation of encapsulated DPSCs in PF-127 hydrogel was detected for 5 days by CCK-8 assay. The results showed that DPSCs could continue to proliferate in PF127 hydrogel from day 1 to day 5, and the difference was statistically significant (Figure 4D).

### 3.5 DPSCs ameliorated the morphological features of the FT tissue

**FIGURE 5:**
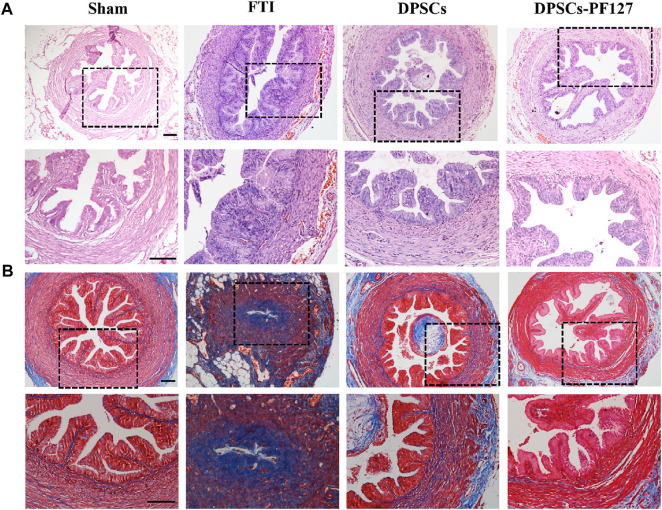
The H&E **(A)** and Masson trichrome **(B)** staining of FT after 4 weeks surgery among the groups. Scale bar: 100 μm.

Four weeks after surgery, upon macroscopic view, injured part of the FT tissue in the FTI and DPSCs groups shrunk greatly (red arrow). In contrast, injured FT tissue in the DPSCs-PF127 group appeared normal and was similar to that of in the sham group (Supplementary Figure S4). Meanwhile, the cross-section of the middle site of the FT tissue was stained with H&E and Masson’s trichrome among the experimental groups. As shown in Figure 5A, the H&E-staining results showed that the normal FT tissue had large mucosa-fold-filled lumen and loosely thick muscularis wall in the sham group. Moreover, the mucosal fold was composed of a single columnar epithelium and a branched core of vascular tissues. In the FTI group, the mucosal fold disappeared, and several infiltrated inflammatory cells and densely thin muscle layers could be observed in the damaged tube tissue. After treatment with DPSCs alone or DPSCs-PF127 *in situ* injection, the FT gradually returned to its normal structure, and mucosal folds with multi-branching recovered, but inflammatory infiltration also occurred in the lumen and muscle wall in the DPSCs group. In the DPSCs-PF127 group, the structure of mucosal folds and muscle walls was almost similar to that of the sham control group. As for Masson’s trichrome staining in each group (Figure 5B), the results showed thin and sparse collagen fibers, which were stained with blue dispersed in the submucosal and muscle wall in the sham group. However, massive collagen fiber infiltration occurred, which was stained with deep blue in the muscle wall and lumen in the FTI group. After treatment with DPSCs alone, a moderate amount of collagen fibers (blue) scattered in the muscle wall, some of which were infiltrated in the lumen. In the DPSCs-PF127 group, the FT preserved its normal structure, and the muscle wall and mucosal fold were filled with a small number of collagen fibers, which had been shown in blue.

### 3.6 Evaluation of TUMEL, immunofluorescence staining, and immunohistochemistry

**FIGURE 6:**
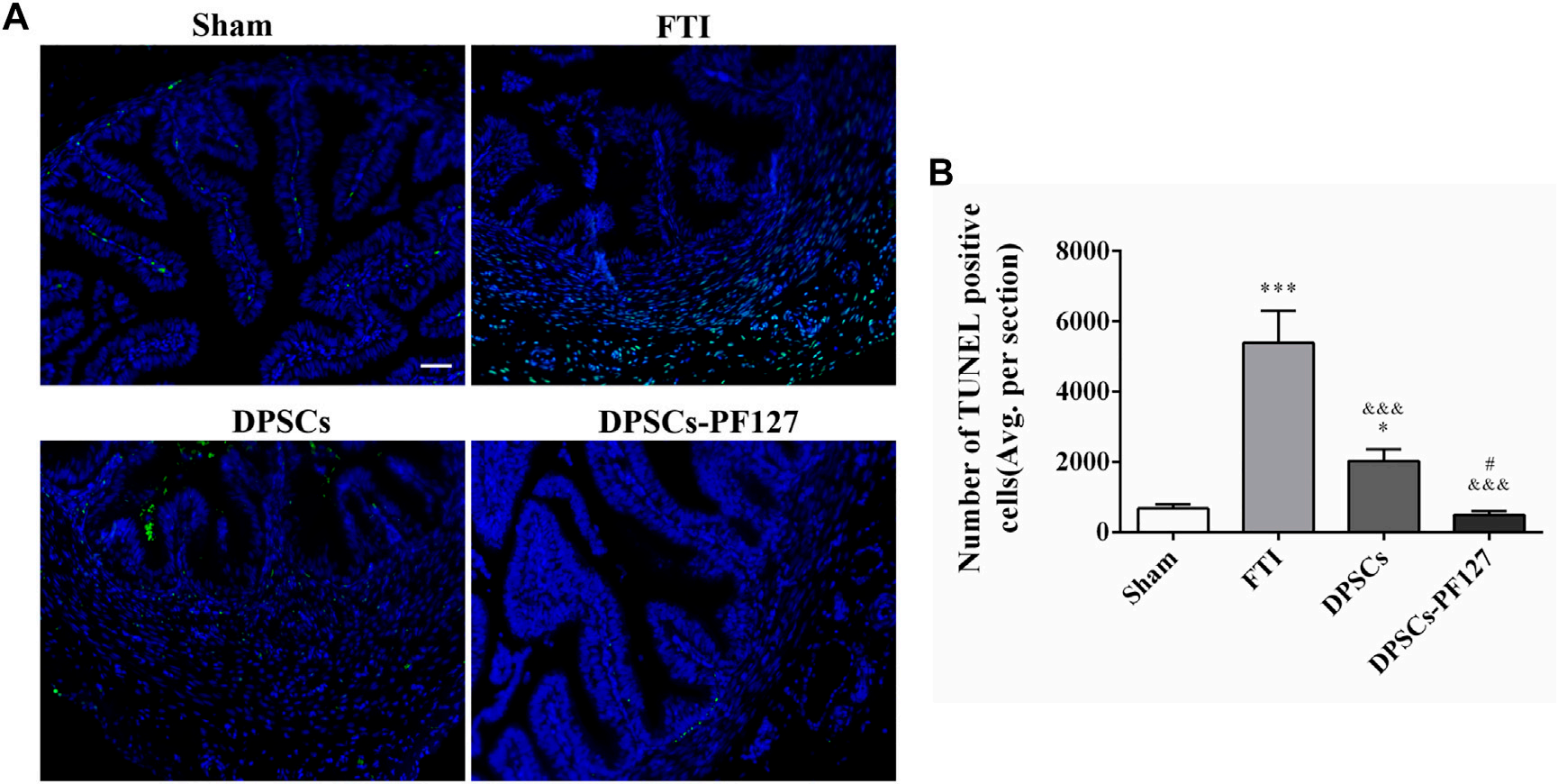
DPSCs-PF127 reduces cell apoptosis of the FT at 4 weeks after injury. **(A)** Immunofluorescence staining for TUNEL (green) of sections from the FT in each group. Scale bar: 50 µm. **(B)** Quantitative estimation of apoptotic and TUNEL cells from three independent sections. All data represent Mean values ±SEM. *p < .05, ***p < .001 versus the Sham group. ^&&&^ *p* < .001 versus the FTI group. ^#^ *p* < .05 versus the DPSCs group.

The effect of DPSCs on FT cell apoptosis was analyzed by TUNEL staining. As shown in Figure 6A, TUNEL staining showed evident green fluorescence in the FTI group, indicating that FT cells underwent significant apoptosis. However, a small amount of TUNEL staining cells were found in the sham group. After treatment of DPSCs, TUNEL staining with green fluorescence gradually reduced, but the expression of TUNEL in the DPSCs group was stronger than that in the DPSCs-PF127 group. The number of TUNEL staining cells was highest in the FTI group and lowest in the DPSCs-PF127 group (Figure 6B). The number of TUNEL staining cells had no significant difference between the sham group and DPSCs-PF127 group (*p* > .05).

**FIGURE 7:**
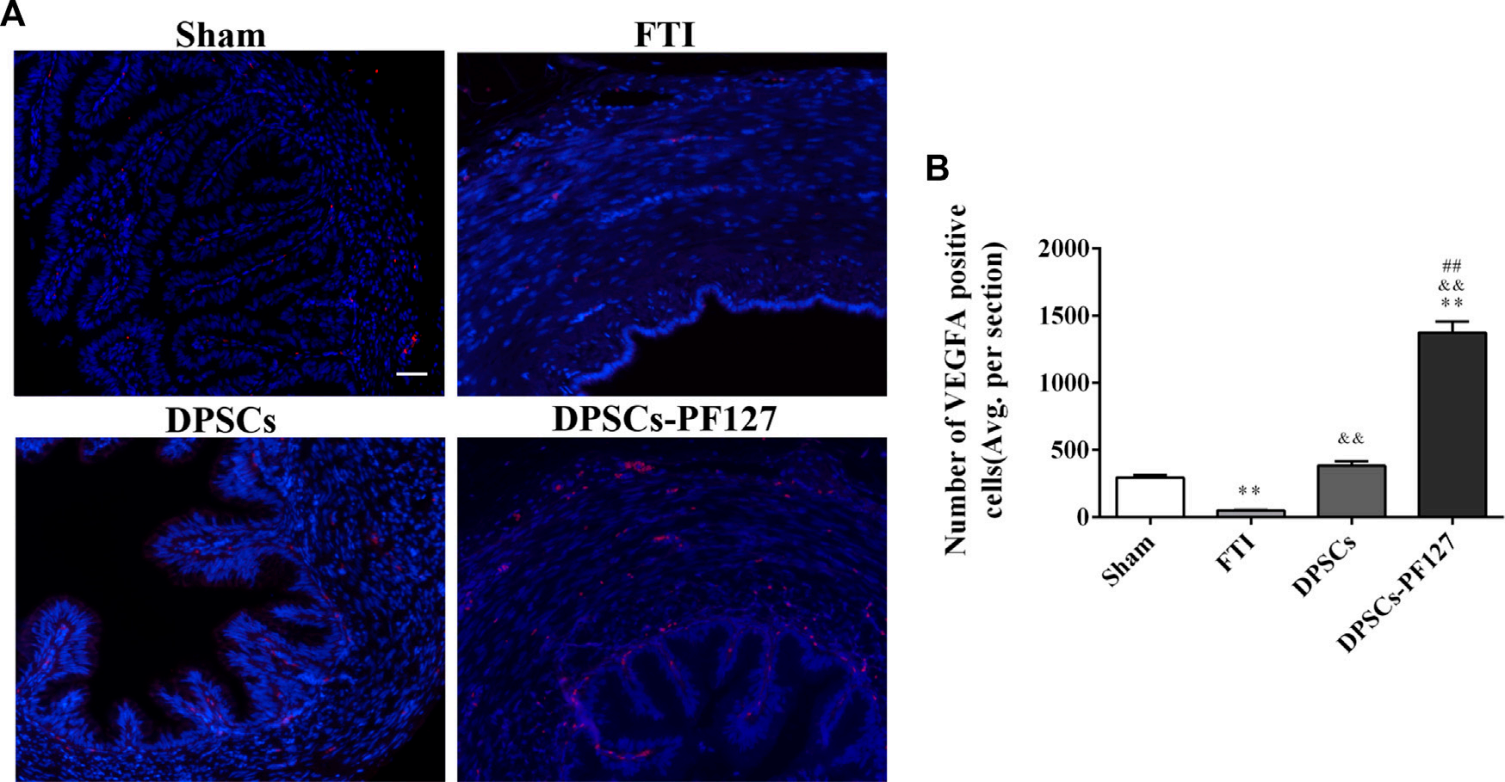
DPSCs-PF127 promotes angiogenesis of the FT at 4 weeks after injury. **(A)** Immunofluorescence staining for VEGF-A (red) of sections from the FT in each group. Scale bar: 50 µm. **(B)** Quantitative estimation of VEGF-A-positive cells from three independent sections. All data represent Mean values ±SEM. **p < .01 versus the Sham group. ^&&^ *p* < .01 versus the FTI group. ^##^ *p* < .01 versus the DPSCs group.

**FIGURE 8:**
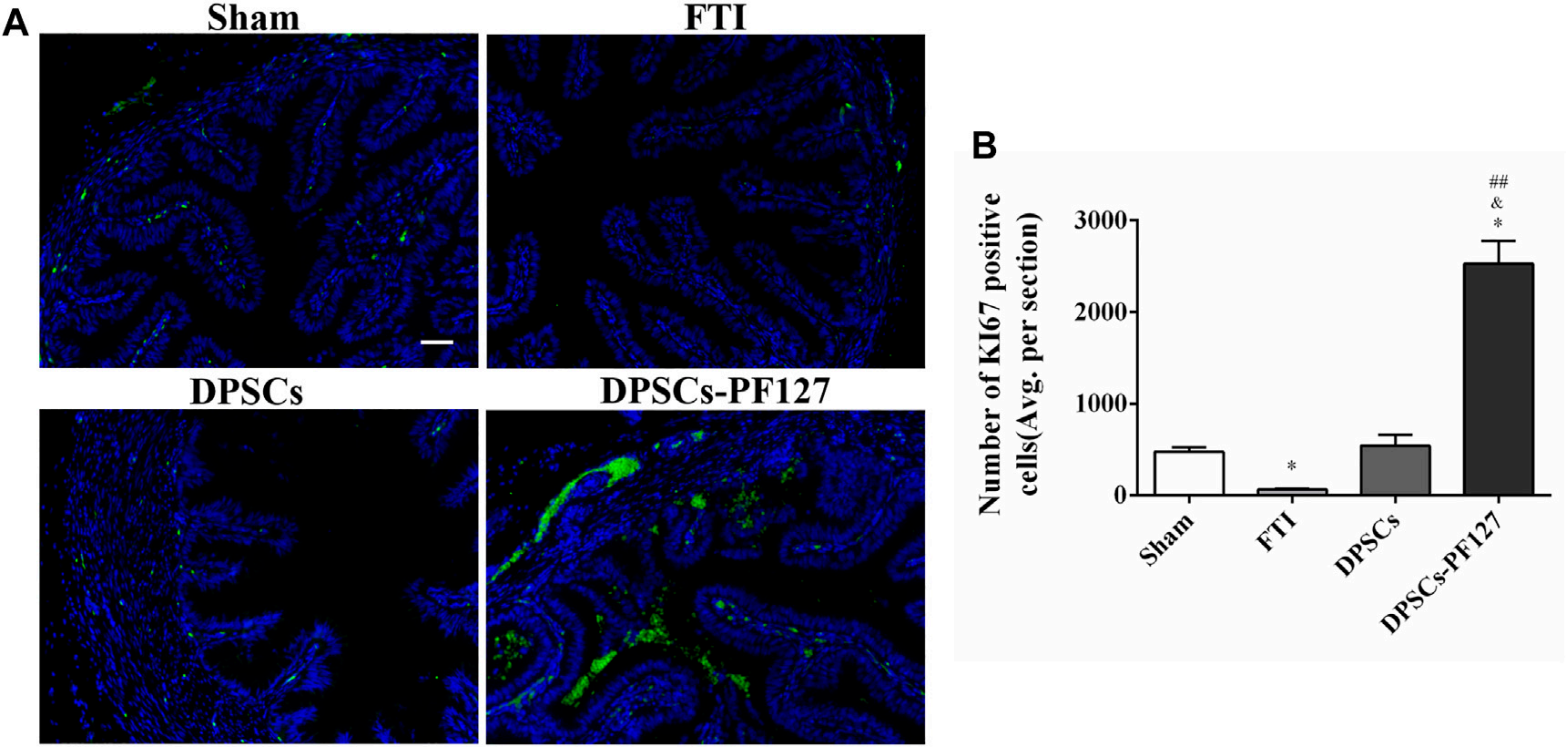
DPSCs-PF127 enhances cell proliferation of the FT at 4 weeks after injury. **(A)** Immunofluorescence staining for KI67 (green) of sections from the FT in each group. Scale bar: 50 µm. **(B)** Quantitative estimation of KI67-positive cells from three independent sections. All data represent Mean values ±SEM. *p < .05 versus the Sham group. ^&^ *p* < .05 versus the FTI group. ^##^ *p* < .01 versus the DPSCs group.

**FIGURE 9:**
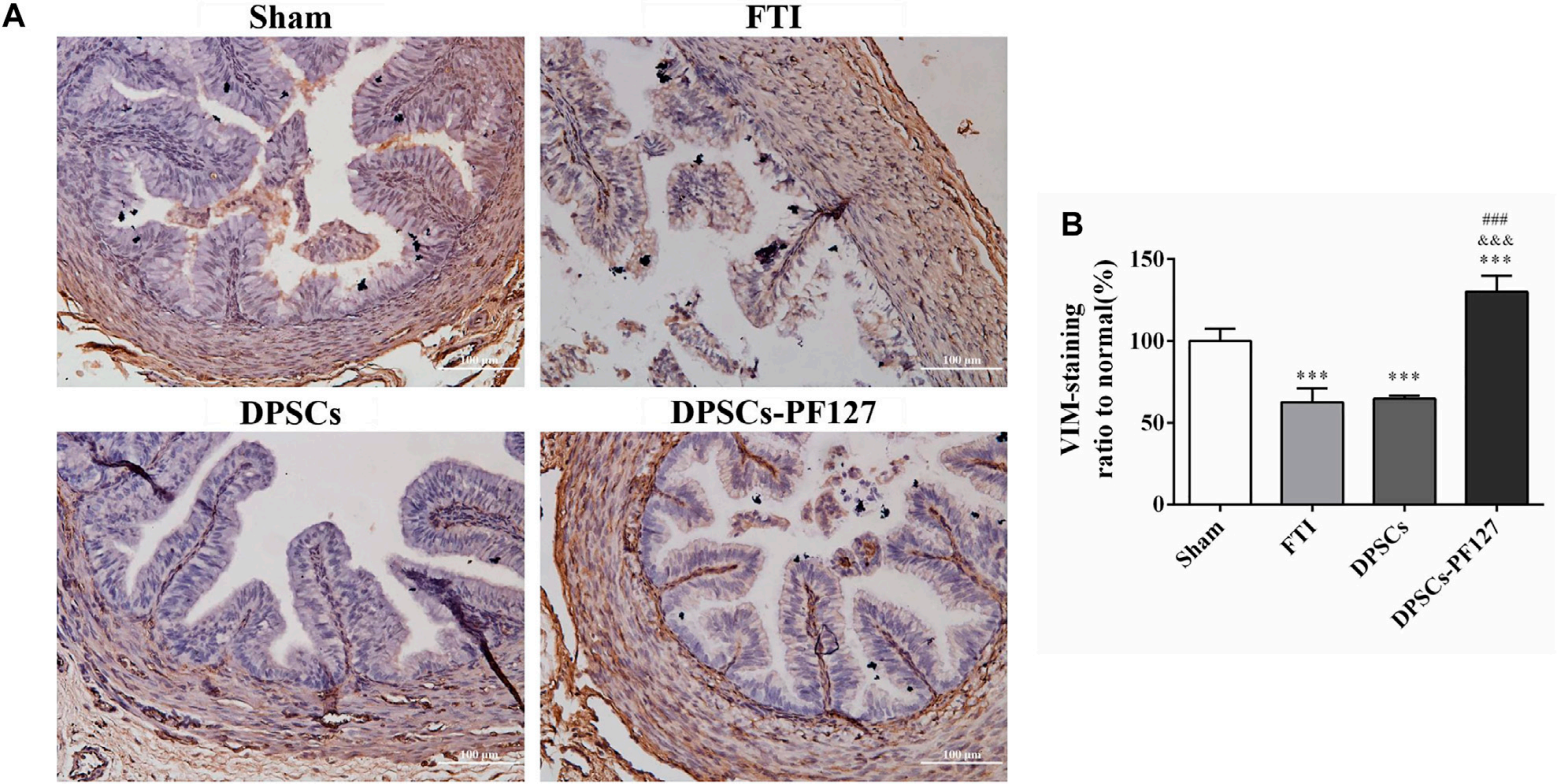
The immunohistochemisty assay of the FT at 4 weeks after injury. **(A)** Representative images of VIM from immunohistochemisty. Scale bar: 100 µm. **(B)** Quantification of the VIM positive staining ratio to normal in FT. The quantification results obtained by ImageJ. All data represent Mean values ±SEM. ***p < .001 versus the Sham group. ^&&&^ *p* < .001 versus the FTI group. ^###^ *p* < .001 versus the DPSCs group.

For immunofluorescence staining and immunohistochemistry analysis, the expression of a cell proliferation marker (KI67) and endothelial cell markers (VEGF-A and VIM) was investigated to evaluate the effect of DPSCs on FT cell proliferation and vascularization *in vivo*. The fluorescence expression of KI67 and VEGF-1 in the DPSCs and DPSCs-PF127 groups was stronger than that in the FTI group. All of the markers were highly expressed in the DPSCs-PF127 group and moderately expressed in the DPSCs and sham groups but weakly expressed in the FTI group (Figures 7A, 8A). In the three experimental groups, the intensity of KI67 and VEGF-A-positive regions was presented in the following order: DPSCs-PF127 > DPSCs > FTI. Meanwhile, a statistically significant difference was found between the DPSCs-PF127 group and the sham group (*p* < .01, Figures 7B, 8B). For immunohistochemistry (Figure 9), the results showed that VIM was highly expressed in the DPSCs-PF127 group and moderately expressed in the sham group but weakly expressed in the DPSCs and the FTI groups (Figure 9A). The intensity of VIM-positive regions was highest in the DPSCs-PF127 group compared with that of the other groups, but no statistically significant difference was found between the DPSCs group and FTI group (*p* > .05, Figure 9B).

### 3.7 Detection of serum TNF-α and IL-10 expression levels and real-time PCR analysis

**FIGURE 10:**
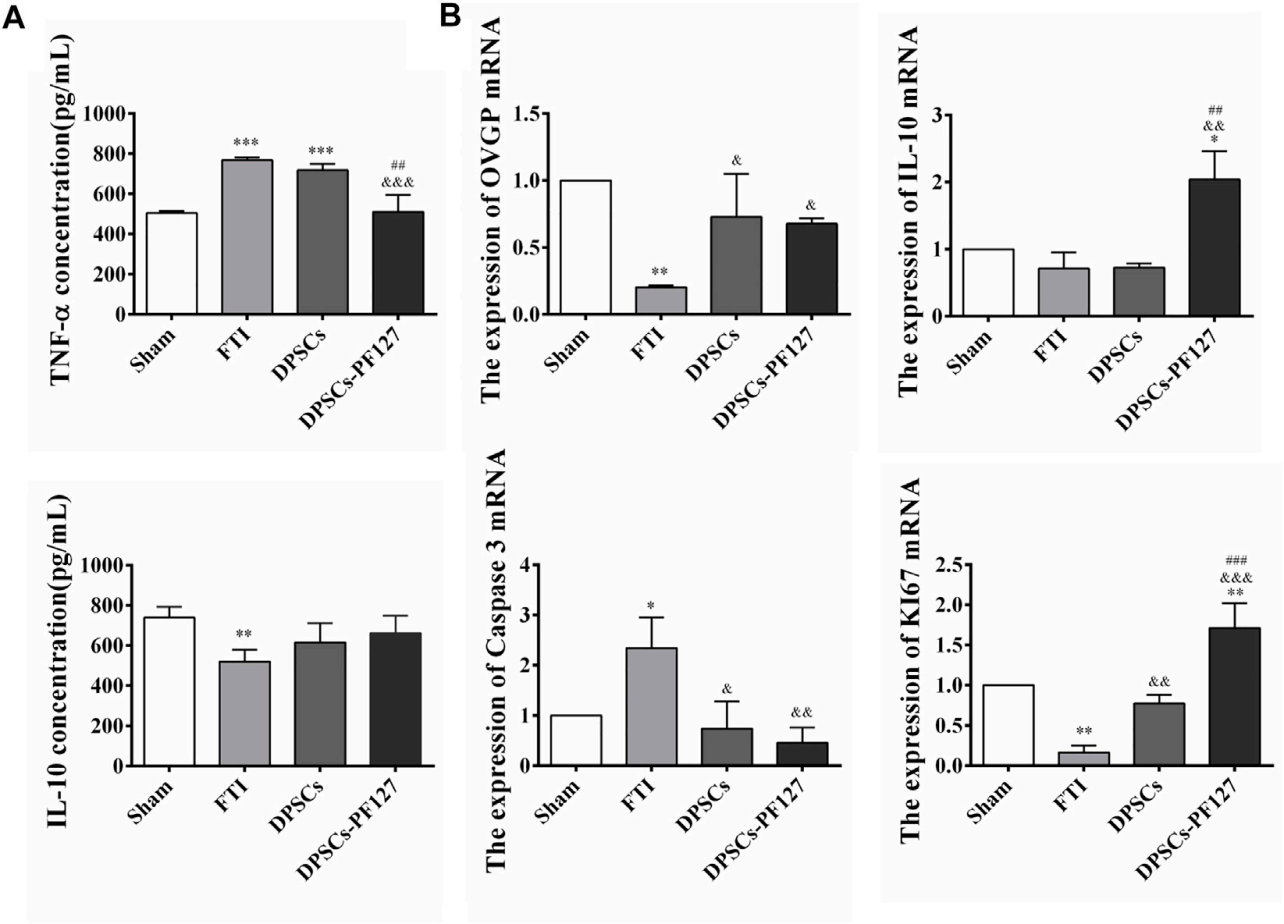
Concentration of inflammatory factors in serum and mRNA detection of the FT at 4 weeks after surgery. **(A)** Comparison of the serum TNF-α and IL-10 concentration among the groups. **p < .01, ***p < .001 versus the Sham group. ^&&&^ *p* < .001 versus the FTI group. ^##^ *p* < .01 versus the DPSCs group. **(B)** The expression of OVGP, IL-10, Caspase 3, KI67 mRNA of the FT tissues among the groups. *p < .05,**p < .01 versus the Sham group. ^&^ *p* < .05, ^&&^ *p* < .01, ^&&&^ *p* < .001 versus the FTI group. ^##^ *p* < .01, ^###^ *p* < .001 versus the DPSCs group.

ELISA was used to evaluate the serum levels of TNF-α and IL-10 in each group. The results showed that the serum TNF-α levels in the FTI and DPSCs groups were significantly higher than those in the sham and DPSCs-PF127 groups (*p* < .001). Meanwhile, no statistically significant difference was observed between the DPSCs-PF127 and sham groups (*p* > .05). However, the serum IL-10 levels in the FTI group were lower than those in the other groups (*p* < .01), and no statistically significant difference was observed among these groups except for the FTI group (*p* > .05, Figure 10A). For real-time PCR (RT-PCR) analysis (Figure 10B), the results indicated that OVGP, IL-10, and KI67 mRNA were highly expressed in the DPSCs-PF127 group and moderately expressed in the DPSCs and sham groups but weakly expressed in the FTI group. However, no significant difference was observed between the sham and DPSCs groups (*p* > .05). In addition, caspase 3 mRNA was weakly expressed in the DPSCs-PF127 group and highly expressed in the TFI group. Moreover, the expression level of caspase 3 showed a significant difference between the TFI group and other groups (*p* < .05).

## 4 Discussion

The FT is important for egg transportation, sperm activation, and early embryonic development in the reproductive process. Therefore, FT, which preserves the normal structure, secretion property, and peristalsis, plays an important role in reproductive functions of women (Lyons et al., 2006; Duran et al., 2014). In addition, the integrity of mucosal fold and the great secretion of epithelial cells in the FT directly affect the surface area of cell reception and cell activation (Hunter 2005). In our previous study, the H&E results confirmed that the structure of FT, particularly the mucosal fold and muscle wall, was destroyed by anhydrous ethanol for 4 h (Supplementary Figure S1C). After 4 weeks, the mucosal fold disappeared, and inflammatory macrophage infiltration, muscle wall fibrosis, and lumen stenosis were observed in the FTI group, which had not received any treatment (Figure 5). Moreover, such structural and functional destructions in the FT would increase the tubal infertility rate. Therefore, the repair and regeneration of the damaged FT played important roles in reducing tubal infertility.

MSCs have shown a strong immunomodulatory property, which could be used as an immunosuppressive agent to modulate the immune response in inflammatory or autoimmune diseases (Liu et al., 2020; Wu et al., 2020; Zhou et al., 2020). In addition, MSCs had a great angiogenic property, and they could promote vessel-like formation by secreting various growth factors or directly differentiating into endothelial cells (Takeuchi et al., 2019; Merckx et al., 2020; Santos et al., 2020). Thus, MSC-based cell therapy, such as Wharton’s jelly-derived mesenchymal stem cells, BM-MSCs, and human umbilical cord mesenchymal stem cells, served as a suitable strategy for the treatment of FT injury. In these studies, most MSCs were used by intraperitoneal or intravenous injection, which had systemic effects on the repair and regeneration of FT injury (Luo et al., 2015; Li et al., 2017; Almasry et al., 2018; Liao et al., 2019). However, no study had reported that the therapeutic method of MSCs in the treatment of FT damage was currently used by *in situ* injection.

In this study, we used DPSCs, which were obtained from dental pulp without harm or invasive surgery, as seeded cells for the treatment of FT injury by PF127 hydrogel *in situ* transplantation. As previously described, PF-127 hydrogel had a thermosensitive property and great cytocompatibility with DPSCs, which was an ideal scaffold for cell survival and proliferation (Albashari et al., 2020). In *in vitro* cell culture experiments, our results indicated that DPSCs, which were dispersed in PF-127 hydrogel, could proliferate from day 1 to day 5 (Figures 4B–D). Moreover, DPSCs showed great immunomodulatory and angiogenic characteristics, and they could promote the expression and secretion of anti-inflammatory cytokines such as IL-10, but they could inhibit the expression and secretion of pro-inflammatory factors such as TNF-α (Figures 1, 2) and enhance the formation of vessel-like structures (Figure 3). Therefore, PF127 hydrogel containing DPSCs could provide an effective treatment for FT injury by *in situ* injection.

For histological analysis, the H&E and Masson’s trichrome staining results indicated that FT structural damage was improved after treatment with DPSCs, but the repair effects in the DPSCs-PF127 group were better than those in the DPSCs group (Figure 5). Previous studies revealed that 3D-network hydrogel could be used as a cell carrier, which could not only ensure the number of active cells in the injured site but also provide a suitable microenvironment for cell growth and proliferation (Long et al., 2020; Villiou et al., 2020). In this study, the results indicated that PF127 hydrogel encapsulating DPSCs by *in situ* injection could be used to promote the repair and regeneration of FT injury.

In our study, TUNEL assay was performed to detect FT apoptotic cells in the injured site because of massive DNA fragmentation in the nuclei of apoptotic cells, which could be labeled with green FITC fluorescence (Zhu et al., 2020; He et al., 2021). Caspase-3, as an important marker of programmed cell death, was responsible for the cleavage of many proteins during apoptosis (Elmore 2007). Our results showed that the positive region of TUNEL staining in the DPSCs-PF127 group was lowest among the groups; meanwhile, caspase-3 mRNA expression was significantly lower in the DPSCs-PF127 group than in the FTI group. These results supported *in situ* transplantation of PF127 hydrogel containing DPSCs into the injured FT, which likely promoted the repair and regeneration of the injured FT by inhibiting cell apoptosis.

KI67, which is a representative proliferation marker, was widely used to evaluate cell proliferation (Willenbacher et al., 2020; Murata et al., 2021). VEGF-A and VIM, which served as vascular endothelial-associated markers contributing to angiogenesis, were commonly used to assess the angiogenic property (Almasry et al., 2018; Chen et al., 2021). In our research, the results revealed that the expression level of KI67, VEGF-A, and VIM was higher in the DPSCs-PF127 group than in the sham control group. Meanwhile, the difference in KI67 and VEGF-A showed no statistically significant difference between the DPSCs and sham control groups, whereas VIM expression showed a contrary result. These results indicated that PF127 hydrogel containing DPSCs could enhance the damage repair and the tissue regeneration of FT, by stimulating the secretion of angiogenic factors and promoting cell proliferation at the injury site.

Tumor necrosis factor-alpha (TNF-α) and interleukin-10 (IL-10) are two representative cytokines produced by the immune cells. Both play key roles in maintaining immune homeostasis and modulate the inflammatory reaction (Wang et al., 2022). And IL-10 can inhibit the production of TNF-α (Kuwata et al., 2003; Passoja et al., 2010). Thus, in this study, TNF-α and IL-10 were used to evaluate the immunomodulatory function of DPSCs in the repair of injured FT. TNF-α, which is a pro-inflammatory factor produced by mononuclear macrophages, was closely associated with the reproductive immunization of the female genital system (Morales et al., 2006; Oróstica et al., 2013). IL-10, which served as a cytokine exerting anti-inflammatory effects, was produced by activated macrophages. A previous study indicated that DPSCs could upregulate the secretion of IL-10 by coculturing with T cells (Demircan et al., 2011). Our results showed that the expression of IL-10 in serum had no significant difference between the DPSCs-PF127 and sham control groups. Moreover, the mRNA expression of IL-10 in the DPSCs-PF127 group was higher than that in the sham control group. However, the level of TNF-α showed a lower expression in the DPSCs-PF127 group than in other groups. These results indicated that PF127 hydrogel containing DPSCs promoted the repair and regeneration of FT damage, thereby stimulating the secretion of IL-10 and inhibiting the secretion of TNF-α.

Oviductal glycoprotein (OVGP), which is a multi-protein complex produced by the secretion of FT epithelium, could be used to evaluate the function of epithelial cells (Luo et al., 2015; Li et al., 2017). Our results indicated that OVGP was highly expressed in the DPSCs and DPSCs-PF127 groups, which had no significant difference compared with the sham control group. In addition, OVGP was weakly expressed in the FTI group, indicating a serious damage in the mucosal fold. These results indicated that DPSCs could promote the repair and regeneration of mucosal fold and restore its secretion functions.

## 5 Conclusion

In the present study, the effects of PF127 hydrogel containing DPSCs on the repair and regeneration of FT injury were evaluated by histological analysis (e.g., H&E and Masson’s trichrome staining, TUNEL staining, immunofluorescence staining, and immunohistochemistry), ELISA, and RT-PCR detections. Our results indicated that PF127 hydrogel containing DPSCs promoted the repair and regeneration of FT damage, thereby inhibiting cell apoptosis, stimulating the secretion of angiogenic factors, promoting cell proliferation, modulating the secretion of inflammatory factors, and restoring the secretion of epithelial cells. Therefore, this study was the first to report that *in situ* injection of PF127 hydrogel encapsulating DPSCs could be used to treat FT injury in clinical settings.

## Data Availability

The original contributions presented in the study are included in the article/Supplementary Material, further inquiries can be directed to the corresponding authors.
