# Supplementary material for: Application of thermosensitive-hydrogel combined with dental pulp stem cells on the injured fallopian tube mucosa in an animal model
Source: Front Bioeng Biotechnol. 2023 Jan 6;10:1062646. doi: 10.3389/fbioe.2022.1062646 (PMC9852820; doi:10.3389/fbioe.2022.1062646)
Supplement: Supplementary file 1 [file DataSheet1.docx]

Supplementary Material

## Supplementary Figures

##
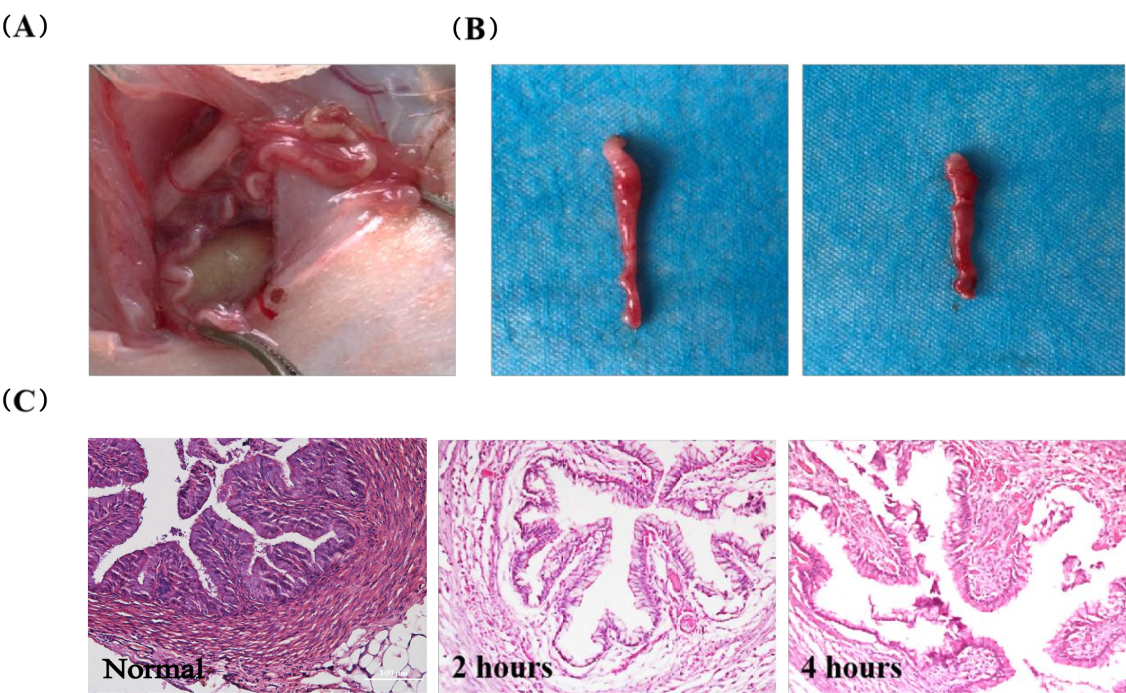


## **Supplementary Figure 1.** Macroscopic appearance and H&E staining (X200) of FT. **(A)** Rough view of rabbit FT. **(B)** Rough view of FT 2 hours and 4 hours after injection of absolute ethanol. **(C)** H&E staining of FT in the normal group and model group.


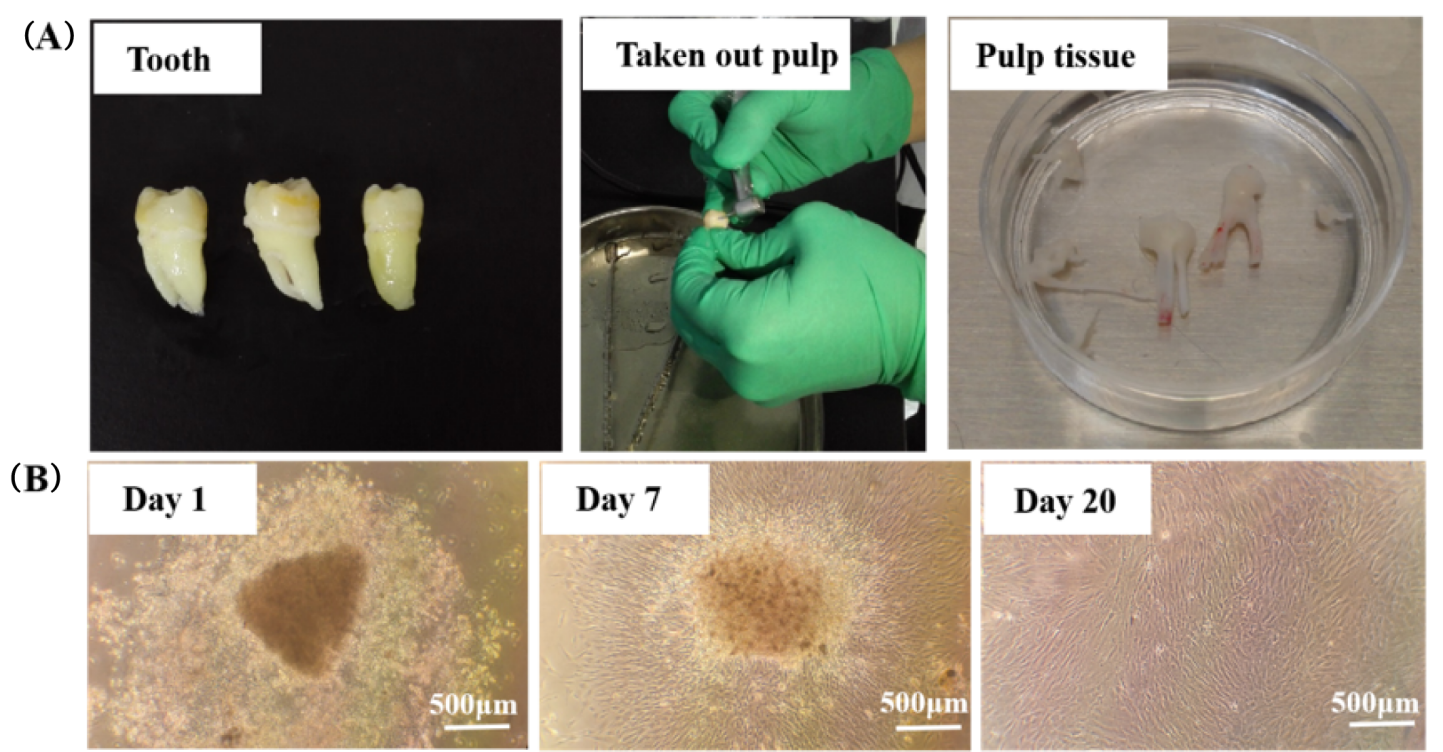


**Supplementary Figure 2.** The procedure of obtaining the dental pulp tissues **(A)** and the primary culture of the dental pulp stem cells **(B)**


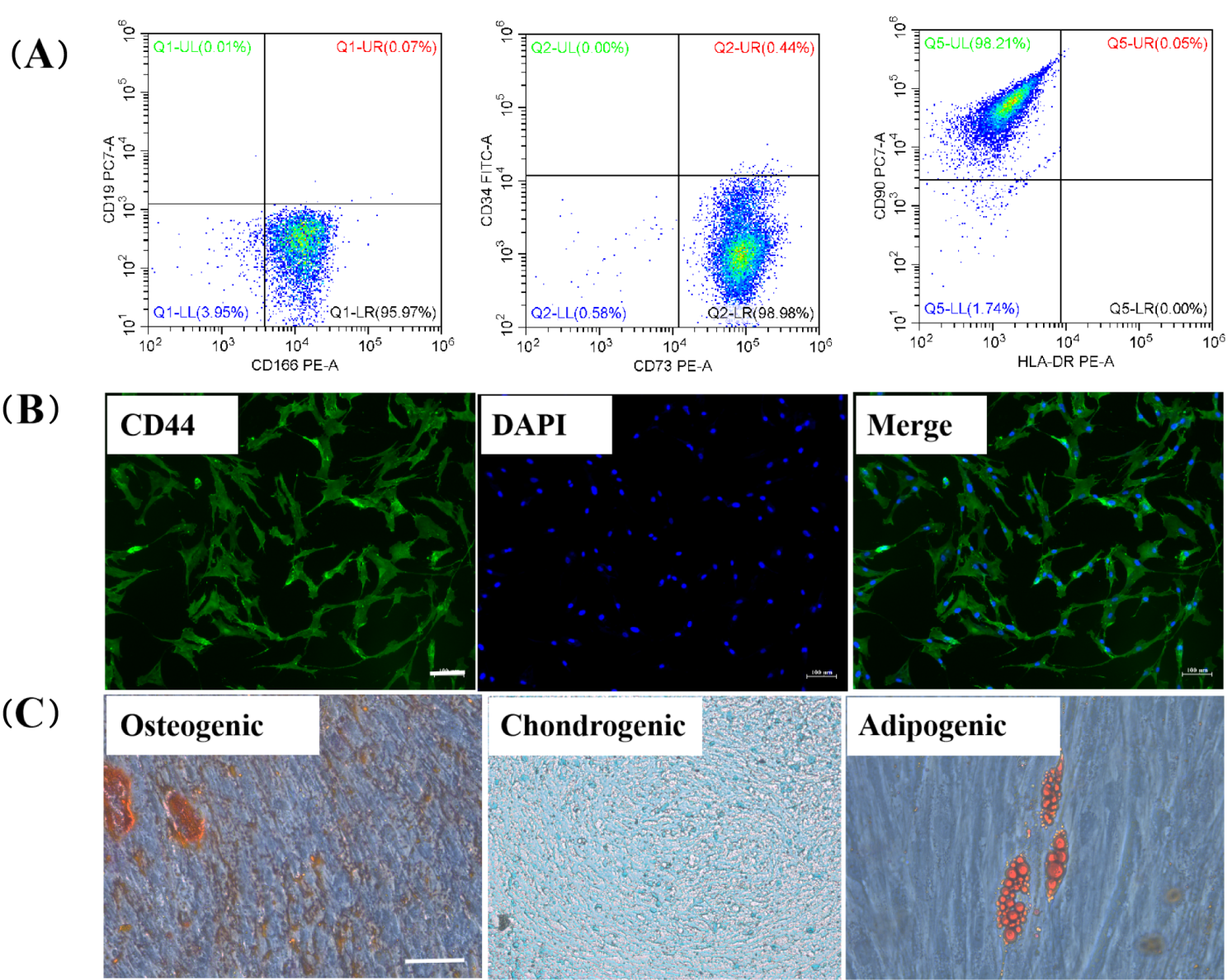


**Supplementary Figure 3.** The identification of DPSCs. **(A)** The expression of MSCs-like surface markers (CD166, CD19, CD73, CD34, CD90) was evaluated by flow cytometry. **(B)** The expression of MSCs-like surface markers CD44 was analysed by immunofluorescence staining. Scale bar: 100 μm. **(C)** The osteogenic/chondrogenic/adipogenic differentiation potential of DPSCs. Scale bar: 50 μm.


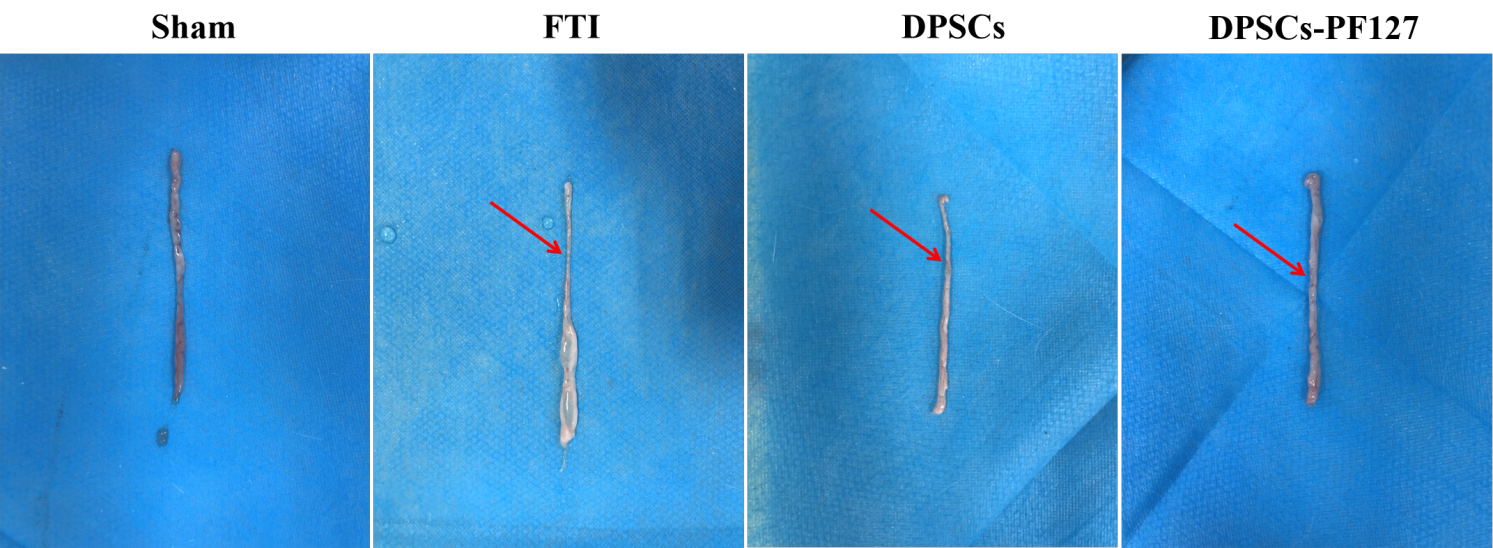


**Supplementary Figure 4.** The light microscopic photograph of the whole FT tissue after surgery for 4 weeks (Red arrow: the injured site).


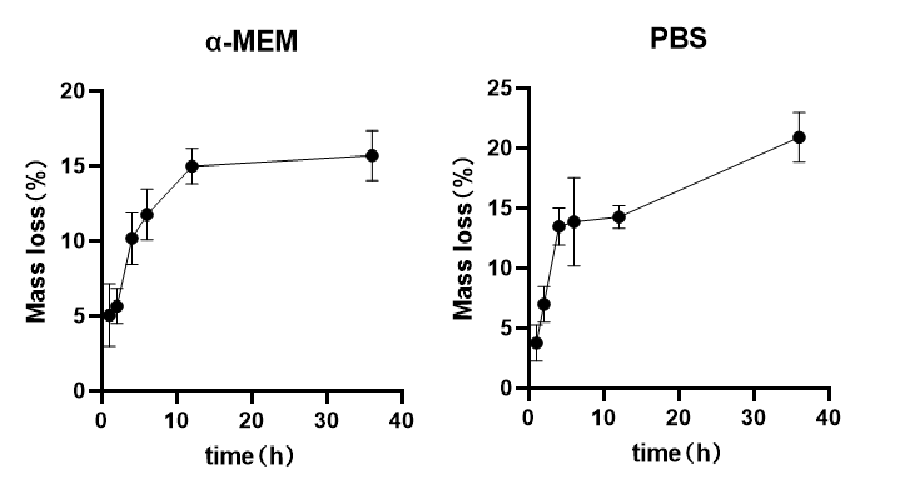


**Supplementary Figure 5.** The in vitro degradability of PF127 hydrogel. Degradation profile of PF127 hydrogel incubated in complete α-MEM (containing 20% FBS, 100 μg/mL of penicillin, and 100 μg/mL of streptomycin) and PBS at 37 °C for 36 hours.
